# A Rare Presentation of Hypocalcaemia-Induced Seizure Secondary to Late-Onset Post-Thyroidectomy Complication

**DOI:** 10.7759/cureus.84167

**Published:** 2025-05-15

**Authors:** Yameen Hamid, Md Al Amin Sarkar, Sudip Karmaker, Samarea Nowrin, SM Amanat Ullah

**Affiliations:** 1 Stem Cell and Translational Neurology, University of Edinburgh, Edinburgh, GBR; 2 Acute Medicine, Medway NHS Foundation Trust, Gillingham, GBR; 3 General Practice, Maidstone and Tunbridge Wells NHS Trust, Maidstone, GBR

**Keywords:** endocrinology, hypocalcaemia induced seizure, hypocalcaemia without hypoparathyroidism, late-onset hypocalcaemia, post-thyroidectomy complication

## Abstract

We report a rare case of late-onset hypocalcaemia presenting with seizure in a female patient, occurring approximately 25 years after a total thyroidectomy. Initial investigations ruled out intracranial pathology, and biochemical analysis confirmed severe hypocalcaemia. The patient responded to intravenous calcium gluconate infusion, with subsequent stabilisation on oral calcium therapy. Notably, the patient had been non-compliant with long-term calcium supplementation. This report underscores that chronic hypocalcaemia can manifest with acute neurological symptoms such as seizures and highlights the importance of long-term follow-up for post-thyroidectomy patients.

## Introduction

Hypocalcaemia is the most common complication following thyroidectomy, occurring in 7-48% of patients post-thyroid surgery [1]. The majority, ranging from 19-38% of patients, experience transient post-surgical hypoparathyroidism, which typically resolves within six months. Hypocalcaemia presenting six months post-surgery is seen in around 3.6% of cases, while permanent hypocalcaemia is observed in approximately 1-5% of patients [1,2]. Hypocalcaemia-induced seizure is more common in children than adults, and the incidence in adults is not reported [3].

Post-surgical hypoparathyroidism leading to hypocalcaemia poses a persistent clinical challenge for thyroid surgeons, primarily due to its frequent occurrence and the lack of well-defined preoperative predictors. However, some identified risk factors of post-thyroidectomy hypocalcaemia are female gender, lymph node dissection, type of thyroidectomy, extent of thyroidectomy, length of procedure, performing cervical lymphadenectomy, re-intervention in cases of persistence or recurrence of thyroid carcinoma or goitre and second intervention for bleeding after surgery. The risk is increased by preoperative low calcium, low parathormone (PTH), and low 25-hydroxy vitamin D [4]. The primary mechanisms underlying hypoparathyroidism after thyroidectomy include vascular disruption, thermal or electrical injury, and mechanical damage, which may result in partial or complete removal of the parathyroid glands [5]. Administering prophylactic calcium and vitamin D supplements when hypocalcaemia is anticipated can effectively prevent the onset of hypocalcaemia [6].

Calcium plays a critical role in both neurotransmitter release and muscle contraction. Thus, hypocalcaemia often manifests as a range of neuronal and muscular hyperexcitable clinical features, including seizures, tetany, Chvostek's sign, and bronchospasm [7]. Treatment approaches for hypocalcaemia vary based on the severity and manner in which it presents [8]. We describe a rare case of a 51-year-old female with late-onset post-thyroidectomy hypocalcaemia-induced seizure.

## Case presentation

Clinical presentation

A 52-year-old woman with a history of total thyroidectomy due to papillary thyroid carcinoma was admitted under acute medicine via emergency following one episode of seizure. According to her medical records, she had never been admitted to the hospital with a seizure or severe hypocalcaemia in the past. As witnessed and described by her partner, the seizure had been generalised tonic-clonic. The episode was associated with drooling and teeth clenching, which had lasted for about five minutes and resolved spontaneously without any intervention. She had lost consciousness during the seizure, followed by post-ictal confusion, pain in the facial and jaw muscles, along with a moderate headache.

On the same day, she had felt dizzy and experienced palpitations since morning, and the seizure had occurred in the afternoon. There had been no trigger for the seizure, and she had been sitting in the chair during the incident. There had been no tongue bite, up-rolling movement of eyeball, bowel and bladder incontinence, limb weakness, retrograde amnesia, muscle spasm, paresthesia, or any sensory abnormality. She had also been feeling unwell for three days before admission, which was associated with dysuria and vomiting. She had an unwitnessed seizure-like episode five months before the admission, but she had not sought any medical attention at that time, and there had been no post-ictal complications.

On examination, she was hemodynamically stable, and specific signs of hypocalcaemia were negative, such as Trousseau's sign, nail dystrophy, Chvostek’s sign, or stridor. According to her past medical records, she had undergone total thyroidectomy due to papillary thyroid carcinoma in 1998, followed by iodine radiotherapy. Since then, she had been on levothyroxine due to post-surgical hypothyroidism. However, she was not on any long-term calcium supplement. She could not remember whether she had been prescribed any calcium supplement initially after the surgery, nor did we find any in her general practitioner (GP) record.

Clinical investigations

**Figure 1:**
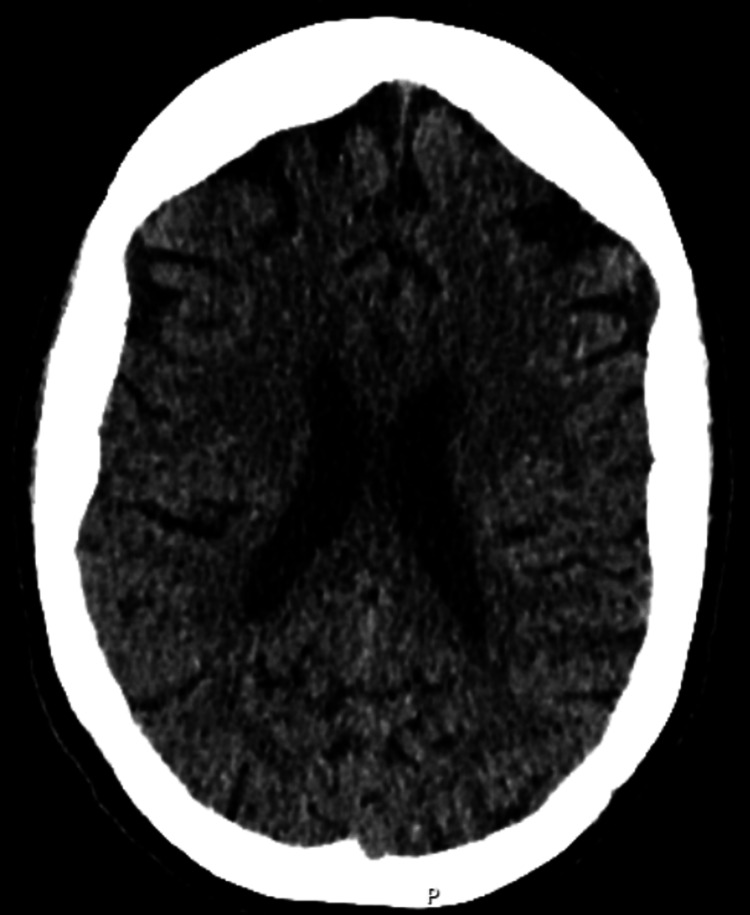
CT brain with no evidence of intracranial lesion or pathology CT: computed tomography

**Figure 2:**
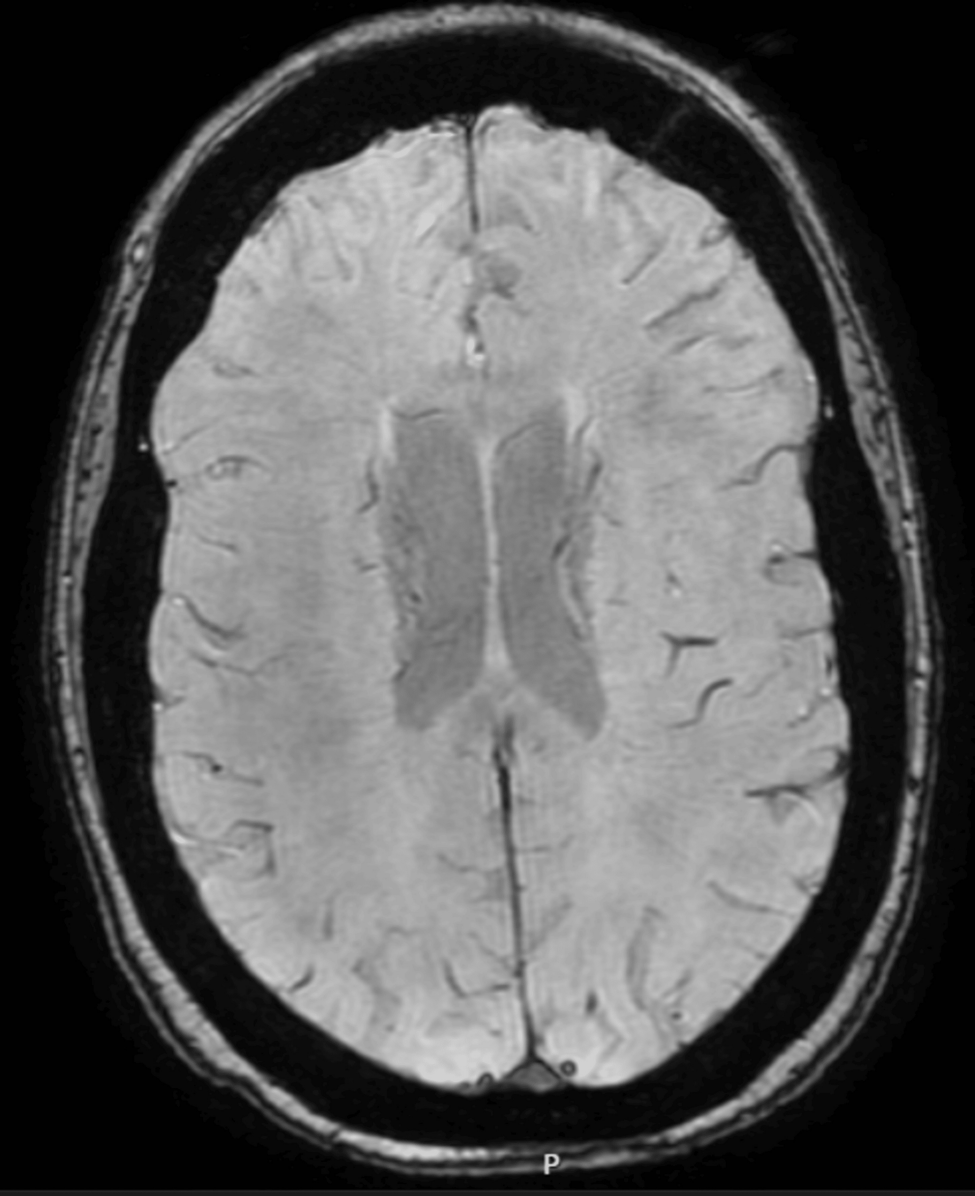
MRI brain with no evidence of intracranial lesion or pathology MRI: magnetic resonance imaging

**Figure 3:**
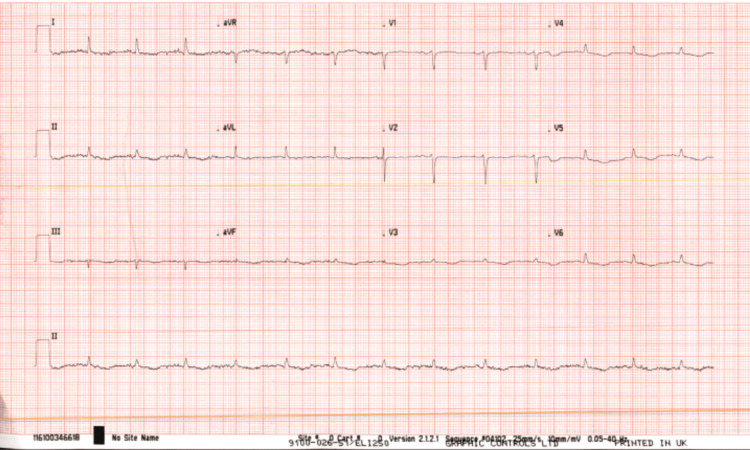
12-lead ECG Precordial leads (V3-V6) show a low-voltage QRS complex. There is no significant QTc prolongation. Atrial fibrillation is present in all leads ECG: electrocardiogram

Her initial calcium level was 1.53 mmol/L (Table 1). Her PTH level was within normal range (19 pg/mL), and she was deficient in vitamin D (Table 2). Serum electrophoresis ruled out any underlying paraproteinemia (e.g., multiple myeloma). As the patient reported dysuria and vomiting, the bedside urine dipstick test was performed, which was positive for nitrate and leukocyte. However, the urine sample sent for culture and sensitivity testing did not grow any microorganism. No abnormality was detected on CT and MRI of the brain (Figures 1, 2). In the ECG, atrial fibrillation and low-voltage QRS were detected in some precordial leads; there was no significant QTc prolongation (Figure 3). 

**Table 1:** Calcium level progression with intravenous calcium therapy Reference value: adjusted calcium: 2.20-2.60 mmol/L

	Day 0	Day 1	Day 2	Day 3	Day 4	Day 5	Day 6	Day 7	Day 8
Adjusted calcium (mmol/L)	1.53	1.70	1.86	1.93	2.02	1.91	1.93	2.46	2.45

**Table 2:** Relevant biochemical markers to exclude differential diagnosis *Standard range is obtained from North Kent Pathology Service (NKPS) eGFR: estimated glomerular filtration rate; PTH: parathormone

Biochemical marker	Value	Reference value^*^
PTH	19 pg/ml	10-65 pg/ml
Serum albumin	33 g/L	35-50 g/L
Serum magnesium	0.72 mmol/L	0.7-1.0 mmol/L
Serum phosphate	1.13 mmol/L	0.8-1.5 mmol/L
25 (OH) vitamin D	19 nmol/L	Deficiency: <25 nmol/L
Creatinine	46 μmol/L	45-84 μmol/L (female)
eGFR	>90 mL/min/1.73 m^2^	Normal: >90 mL/min/1.73 m^2^

Treatment and follow-up

The patient's calcium level improved to 1.70 mmol/L on the following day after infusing IV 10% calcium gluconate in a 10 ml solution over 10 minutes. The IV calcium gluconate infusion was continued for four more days, which improved her calcium level to 2.02 mmol/L. As her calcium level again dropped to 1.91 mmol/L on the fifth day, 10% calcium gluconate in a 100 ml solution was infused again for two days. Subsequently, calcium level improved to 2.46 mmol/L, which remained stable (Table 1); she had been seizure-free since her admission to the hospital. She was discharged with regular and long-term combined oral calcium and vitamin D supplements (calcium carbonate 1500 mg/colecalciferol 400 unit tablet twice a day). She attended the Endocrinology clinic for a follow-up two months later, and her calcium level was found to be 2.16 mmol/L while she was on regular long-term calcium replacement therapy.

## Discussion

We presented a rare case of late-onset post-surgical hypocalcaemia-induced seizure. Though post-thyroidectomy hypocalcaemia is often encountered in clinical settings, only 19 cases of late-onset post-surgical hypocalcaemia have been reported [9], and even fewer cases of late-onset post-surgical hypocalcaemia-induced seizure [10]. One similar case to ours was reported by Arpaci et al., which involved a 63-year-old female with generalised seizure due to hypocalcaemia about 20 years after thyroid surgery [11]. Kamath et al. described a patient with late-onset post-surgical hypocalcaemia-induced seizure who had chronic hypocalcaemic features like muscle spasm and memory loss [12].

Another case of late-onset post-thyroidectomy hypocalcaemia-induced seizure has been reported by Agarwal et al., which was complicated with Parkinsonian symptoms and intracranial calcification [13]. Halperin et al. have published a case series comprising four late-onset post-surgical hypocalcaemia cases, one of which presented with generalised seizure similar to the clinical presentation of the case we described [14]. Post-surgical hypocalcaemia symptoms, i.e., myalgia, confusion, depression, can go unrecognised for many years. Bellamy et al. have described a case where the condition remained undetected for as long as 36 years, which involves the longest duration from surgery to seizure (nearly 34 years) in the literature; in our case, this duration was about 25 years [15]. 

The proposed mechanism of late-onset post-thyroidectomy hypocalcaemia is a gradual decrease in blood circulation of the remaining parathyroid tissue [16]. which could apply to the case we described; however, one major identified risk factor was the fact that the patient had never been prescribed a long-term calcium or vitamin D supplement. High-dose routine supplementation of calcium and vitamin D is a recommended protocol for the prevention of symptomatic hypocalcaemia and readmission [17]. A meta-analysis has provided evidence that combined supplementation of calcium and vitamin D is more efficient than calcium alone in terms of preventing post-thyroidectomy hypocalcaemia [18]. Another important risk factor in our case was female gender, which is also associated with an increased prevalence of post-thyroidectomy hypocalcaemia [19].

The role of calcium in seizures is paradoxical and yet to be understood. Calcium, in general, is responsible for neuronal and neuromuscular junction electrical excitability. Seizure is a neuronal hyperexcitable event which would not be explained by hypocalcaemia. Several studies have shed light on the mechanism where the modulation of other voltage-gated channels may play a role in hyper-excitability induced by hypocalcaemia [7].

The type and mode of thyroid surgery can significantly influence post-thyroid hypocalcaemia; for instance, duration and extension of surgery can increase the risk of hypocalcaemia [4]. In our case, we do not have adequate information regarding the operation, and hence are unable to comment on whether the surgery posed a risk itself. Peri-operative PTH level is a good predictor of post-thyroidectomy hypocalcaemia. Again, in this case, it was not documented, and hence we are unable to comment whether peri-operative PTH could be a risk factor for this patient [20].

Before or after a total or nearly total thyroidectomy, the administration of preoperative vitamin D in patients with pre-operative lower vitamin D levels, with or without calcium, may help with the reduction in the incidence of hypocalcaemia. With preoperative supplementation, there is a tendency towards a decreased need for IV calcium [1]. Our patient could not confirm whether she had been administered preoperative calcium replacement.

## Conclusions

Our objective in reporting this case was to highlight the rarity of late-onset post-surgical hypocalcaemia-induced seizure and raise awareness about the condition among physicians working in emergency and acute medicine. Long-term calcium and vitamin D supplements should be considered a routine protocol in post-thyroidectomy follow-up. This and routine check-up of calcium and PTH levels could easily be performed in primary healthcare settings, such as the general practice milieu in the UK.
